# Convention of the Physicians of N. E. Kentucky

**Published:** 1840

**Authors:** 


### MEETING OF THE PHYSICIANS OF NORTH-EASTERN KENTUCKY.

We have been favored by Dr. Duke of Maysville, with a printed copy of the proceedings of a convention of the physicians of Mason, Bracken and Fleming counties, held at Washington on the 22d of November last. The object of our brethren, in thus coming together, for the first time, seems to have been, to make each others personal acquaintance, to cherish fraternal feelings, and to adopt for their government, as neighboring practitioners, a system of medical ethics. The officers of the convention, were Dr. Nelson, President, Dr's. Johnson and McDowell, Vice-Presidents, Dr. Lawson, Secretary, and Dr's. Sharp, Taliaferro and J. Shackleford a Standing Committee.

Without attaching any very great importance to rules of professional etiquette, which the honorable observe without their being written down, and the dishonorable never hesitate to infringe, although pledged to observe them; we cannot too strongly commend the example set by the medical gentlemen of the north-east corner of our State, and hope to see it imitated by the physicians of other sections of Kentucky, and of the western States generally. Greater professional an dsocial intercourse, than has hitherto existed would contribute to promote the harmony, raise the dignity and augment the influence of the profession; while it could scarcely fail in a short time, to inspire many who now languish in intellect, and loiter in action, with a new spirit of emulation and improvement. The following was one of the most important resolutions adopted by the meeting:

"*Resolved*, That this Association respectfully urge upon the physicians of Kentucky, the expediency of forming district and county societies, for the promotion of medical science; and also, that a State convention be held, in Frankfort, on the second Monday in January, 1841, for the purpose of organizing a State Medical Society."

